# School anxiety profiles in Spanish adolescents and their differences in psychopathological symptoms

**DOI:** 10.1371/journal.pone.0262280

**Published:** 2022-01-21

**Authors:** Aitana Fernández-Sogorb, Ricardo Sanmartín, María Vicent, Carolina Gonzálvez, Cecilia Ruiz-Esteban, José Manuel García-Fernández

**Affiliations:** 1 Department of Developmental Psychology and Didactics, University of Alicante, San Vicente del Raspeig, Alicante, Spain; 2 Department of Evolutionary and Educational Psychology, University of Murcia, Murcia, Spain; University of São Paulo, BRAZIL

## Abstract

School anxiety and psychopathological symptoms tend to co-occur across development and persist in adulthood. The present study aimed to determine school anxiety profiles based on Lang’s model of the triple response system (cognitive anxiety, psychophysiological anxiety, and behavioral anxiety) and to identify possible differences between these profiles in psychopathological symptoms (depression, hostility, interpersonal sensitivity, somatization, anxiety, psychoticism, obsessive-compulsive, phobic anxiety, and paranoid ideation). The School Anxiety Inventory (SAI) and the Symptom Assessment-45 Questionnaire (SA-45) were administered to 1525 Spanish students (49% girls) between 15 and 18 years old (*M* = 16.36, *SD* = 1.04). Latent Profile Analysis identified four school anxiety profiles: Low School Anxiety, Average School Anxiety, High School Anxiety, and Excessive School Anxiety. A multivariate analysis of variance revealed statistically significant differences among the school anxiety profiles in all the psychopathological symptoms examined. Specifically, adolescents with Excessive School Anxiety showed significantly higher levels of the nine psychopathological symptoms than their peers with Average School Anxiety and Low School Anxiety. In addition, the Excessive School Anxiety profile scored significantly higher in phobic anxiety than the High School Anxiety group. These findings allow to conclude that it is necessary enhance well-being and reduce psychopathology of those adolescents who manifest high and very high reactivity in cognitive, psychophysiological, and behavioral anxiety.

## Introduction

The three-dimensional theory of anxiety by Lang [1] determines that there are three response systems of anxiety: cognitive (e.g., worry or unpleasant thoughts of danger), psychophysiological (e.g., muscle tension), and behavioral (e.g., avoidance). Based on this model, school anxiety is conceptualized as a set of cognitive, psychophysiological, and behavioral responses emitted by students who perceive school situations as dangerous or threatening [2].

If students are not properly treated at an early stage, they may experience continuing school anxiety symptoms that often follow a chronic course during adulthood [3]. Adolescents tend to experience school anxiety symptoms with higher frequency as they move on to higher grades throughout the high school years [4]. Therefore, this construct is currently of interest in educational research on adolescence [5]. In this sense, recent studies examined the multidimensionality of school anxiety based on Lang’s theory by using adolescent samples from France [6] and Spain [7]. Students showed different levels of anxiety in the cognitive, psychophysiological, and behavioral responses. Consequently, adolescents could be manifesting different response patterns of school anxiety, characterized by the tendency to show higher anxiety values in the cognitive, the psychophysiological or the behavioral component. It would be possible to identify these response patterns by establishing school anxiety profiles based on Lang’s three-dimensional theory. Thus, the method of latent profile analysis could be used to assign each student to their most suitable group (i.e., latent profile) according to the intensity of their anxiety responses [8]. It would provide relevant scientific information to apply different techniques of treatment depending on the component or components of anxiety that predominate in each group of anxious students [9].

However, only one study of school anxiety profiles was found in previous literature, which was not based on the three components of anxiety. It was conducted in the Spanish adolescent population [10]. Specifically, 365 students aged 12 to 16 (*M* = 13.71; *SD* = 1.47) were grouped into three profiles based on different combinations of common school anxiety-provoking situations (i.e., situations related to aggressive behavior, academic failure, punishment, and social and academic evaluation). These groups were named Low School Anxiety, Average School Anxiety, and High School Anxiety, since its members showed low, moderate, and high levels of school anxiety in all mentioned situations, respectively. In this regard, more research examining school anxiety profiles based on Lang’s model of the triple response system is needed [7], beyond the study of profiles based on school anxiety-provoking situations as it has been done so far in literature [10].

Anxious students tend to manifest mental health problems, both internalizing and externalizing [11–14]. In fact, responding anxiously to situations from early adolescence suppose a risk factor for the development of psychopathological problems [15]. Thus, several works have examined the co-occurrence of anxiety with psychopathological symptoms in the adolescent population. Specifically, these studies obtained a positive and significant relationship of anxiety with depression [16–18] and obsessive-compulsive symptoms [16, 18–20]. Regarding the sample’s characteristics, participants (12–18 years old) from European or Asian countries were recruited for conducting these works. Only two studies used clinical samples of adolescents with obsessive–compulsive disorder or anxiety disorders [18, 20].

As regards the relationship between school anxiety, understood as a multidimensional construct with three response systems, and psychopathology, one study was performed in the adolescent population [21]. A sample of 1409 Spanish students aged 12 to 18 (*M* = 14.32; *SD* = 1.88) was recruited for examining the correlations of school anxiety with depression, trait anxiety (i.e., an emotional tendency to react anxiously to situations perceived as dangerous), and state anxiety (i.e., a transitory emotional state manifested in a specific situation). Positive and significant correlations were found between each response system of school anxiety (i.e., cognitive anxiety, psychophysiological anxiety, and behavioral anxiety) and depression, trait anxiety, and state anxiety.

To sum up, anxiety has proved to be related to a reduced number of internalizing (depression, trait anxiety, and state anxiety) and externalizing (obsessive-compulsive symptoms) problems during adolescence. In this regard, other psychopathological characteristics that could be affecting the development of anxious students who refuse school were recently analyzed on a sample of 1894 Spanish adolescents aged 15 to 18 (*M* = 16.84; *SD* = 1.03) [22]. The participants responded to the Spanish version of the Symptom Assessment-45 Questionnaire (SA-45) [23], which is widely used for establishing the level of severity of psychopathology in community and clinical settings. Specifically, the SA-45 provides a broad overview of psychopathology across several basic domains, including depression, hostility, interpersonal sensitivity, somatization, anxiety, psychoticism, obsessive-compulsive, phobic anxiety, and paranoid ideation [24]. The authors obtained that those Spanish students who resisted attending school because of anxiety and positive reinforcements, in comparison with those participants with low school refusal behavior, showed a significantly higher tendency to manifest all nine symptoms of psychopathology [22]. Therefore, they found that not only depression or obsessive-compulsive symptoms are linked to anxiety among the adolescent population, but also other basic psychopathological domains, such as hostility or somatization, appear to be related to school refusal behavior based on anxiety and positive reinforcements.

Despite the high level of severity of psychopathology manifested by anxious students who refuse school in Spain, it is currently unknown whether the Spanish adolescents who differ in their cognitive, psychophysiological, and behavioral responses of school anxiety tend to manifest different levels of psychopathological symptoms. Thus, all these basic psychopathological domains that are particularly comorbid with anxiety should be examined in relation to school anxiety responses to broaden knowledge on mental health issues that affect students’ development and learning [25]. Moreover, relationships between school anxiety responses and psychopathological problems could be better understood by previously establishing school anxiety profiles based on different combinations of the three response systems. It would allow education professionals to design specific interventions adapted to the response pattern of school anxiety and to the severity of internalizing and externalizing symptoms manifested by each group of students [26].

To overcome the previous shortcomings, the general aim of this study was to examine the relationship between school anxiety and the psychopathological symptoms assessed by the SA-45 in a sample of Spanish adolescent students. The specific objectives were: (a) to identify school anxiety profiles based on Lang’s model of the triple response system, and (b) to verify whether the students grouped into school anxiety profiles differed significantly in their levels of depression, hostility, interpersonal sensitivity, somatization, anxiety, psychoticism, obsessive-compulsive, phobic anxiety, and paranoid ideation.

Considering the literature review, we expected that:

*Hypothesis 1*: the combinations of the response systems (i.e., cognitive anxiety, psychophysiological anxiety, and behavioral anxiety) would result in different school anxiety profiles.
*Hypothesis 2*: the school anxiety profile with the highest levels of cognitive, psychophysiological, and behavioral responses would show significantly higher scores in all the psychopathological symptoms examined, since the previous study [22] found that Spanish students with the highest levels of school refusal behavior based on anxiety and positive reinforcements showed the highest scores in all nine psychopathological domains.

## Materials and methods

### Participants

The sample was recruited by random sampling by conglomerates. Five geographical areas (north, south, east, west, and center) of two Spanish provinces (Alicante and Murcia) were considered. One or two secondary education centers were selected in each geographical area, with 17 being the total number of public, concerted, and private centers chosen. Then, four classrooms per center were randomly selected.

The initial sample consisted of 1710 students, of which 102 (6%) were excluded for not having written consent from their parents or legal guardians and 83 (4.9%) were excluded because they completed the self-report measures with omissions or errors (i.e., providing more than one answer per item). As a result, the final sample comprised 1525 participants between 15 and 18 years old (*M* = 16.36, *SD* = 1.04). Table 1 presents the frequency distribution by gender and age. The chi-square test revealed a uniform distribution of the sample (χ^2^_(3)_ = 3.75, *p* = 0.29).

**Table 1:** Sample distribution by gender and age.

Gender	Age				Total
	15	16	17	18	
Boys (%)	183 (12%)	242 (15.9%)	212 (13.9%)	140 (9.2%)	777 (51%)
Girls (%)	206 (13.5%)	213 (14%)	205 (13.4%)	124 (8.1%)	748 (49%)
Total	389 (25.5%)	455 (29.9%)	417 (27.3%)	264 (17.3%)	1525 (100%)

### Measures

The *School Anxiety Inventory* (SAI [27]) is a self-report instrument designed to assess school situations and responses of anxiety in the adolescent population. It consists of four situational factors: anxiety about academic failure and punishment (8 items), anxiety about aggression (6 items), anxiety about social evaluation (5 items), and anxiety about academic evaluation (4 items). In addition, it has three response systems: cognitive anxiety (9 items), psychophysiological anxiety (5 items), and behavioral anxiety (5 items). Items are answered on a five-point scale (0 = never; 4 = always). The SAI proved adequate psychometric properties in its validation study with Spanish adolescents [27]. In this study, we only used the three factors referred to response systems of anxiety, whose Cronbach’s alpha coefficients were: cognitive anxiety (.83), psychophysiological anxiety (.85), and behavioral anxiety (.83).

The *Symptom Assessment-45 Questionnaire* (SA-45 [28]) is derived from the Symptom Check List-90 (SCL-90 [29]). The SA-45 is a self-report measure that assesses psychopathological symptoms in both general and clinical populations. Specifically, its 45 items are grouped along nine 5-item scales: depression, hostility, interpersonal sensitivity, somatization, anxiety, psychoticism, obsessive-compulsive, phobic anxiety, and paranoid ideation. A five-point Likert scale (0 = not at all; 4 = very much or extremely) is used for reporting how frequently individuals experienced each of the 45 psychopathological symptoms during the last week. We used the Spanish version of the SA-45, which showed optimal psychometric properties in its validation study [23]. In the present work, adequate reliability values were also obtained: depression (.75), hostility (.80), interpersonal sensitivity (.77), somatization (.80), anxiety (.77), psychoticism (.71), obsessive-compulsive (.73), phobic anxiety (.80), and paranoid ideation (.70).

### Procedure

In the first place, the principal of each educational center was interviewed. In these meetings, the research aims were presented, and principals’ permission was requested. Then, a letter was sent to the participants’ legal tutors to inform them about the characteristics of the research. This letter was signed by those legal guardians who agreed to participate and was added to the documentation of the project approved by the ethics committee. The questionnaires were collectively administered during the school day, having an average administration time of 20 minutes for the SAI and 15 minutes for the SA-45. A member of the research team was always present to explain the procedure, solve doubts, and ensure students that their participation was voluntary and anonymous. Ethical standards of the 1964 Declaration of Helsinki were followed, and the research protocol was approved by the Ethics Committee of the University of Alicante (File number: 20170905).

### Data analysis

Latent Profile Analysis (LPA) was performed to determine the number of school anxiety profiles. The standardized *z* scores obtained in the response system factors were used. These z scores were interpreted according to the following criteria: *z* scores below -.5 indicated low levels of school anxiety, *z* scores between -.5 and .5 showed moderate levels of school anxiety, and *z* scores over .5 indicated high levels of school anxiety [30]. The optimal profile solution that best fitted to data was determined considering several fit indices [31]: the lowest values of the Akaike Information Criteria (AIC) and Bayesian Information Criteria (BIC); entropy values close to 1; and *p*-values below to .05 for the Vuong-Lo-Mendell-Rubin Likelihood-Ratio Test (LRT) and the Bootstrap Likelihood Ratio Test (BLRT). In addition, the size of the profiles was also considered to choose the best model, since each profile should be represented by at least 1% of the sample [32]. Finally, the interpretability and psychological significance of each solution was examined according to previous scientific literature [33].

The differences in the psychopathological symptoms among the school anxiety profiles were analyzed by a multivariate analysis of variance (MANOVA). The eta square index (*η*^*2*^) and post hoc tests (Bonferroni method) were performed to determine between which profiles there were statistically significant differences. Furthermore, Cohen’s *d* index was calculated to obtain the magnitude of these differences. According to Cohen’s criteria [34], *d*-values between .20 and .49 represent low effect sizes, *d-*values between .50 and .79 represent moderate effect sizes, and *d*-values above .80 represent large effect sizes.

Data were analyzed using SPSS version 26 and MPlus version 8 programs.

## Results

### School anxiety profiles

Five different solutions with a progressive number of groups were analyzed. The fit indices of each model are displayed in Table 2. The AIC and BIC values decreased progressively for each solution that increased one group with the six-profile model having the lowest AIC and BIC values. On the other hand, the three-profile model had the entropy value closest to 1. However, none of these solutions had a *p*-value below to .05 for the LRT, and five- and six-profile solutions included one and two groups that did not achieved 1% of the sample, respectively. On the basis of available data, the four-profile model was deemed the most parsimonious solution with *p* < .05 for the LRT and BLRT, the third lowest AIC and BIC values, an entropy value close to 1, and all profiles having achieved at least 1% of the sample.

**Table 2:** Model fit indices for the five profile solutions of the Latent Profile Analysis (LPA).

Model	AIC	BIC	Entropy	LRT	BLRT	Size
2 profiles	11508.24	11561.53	.82	.00	.00	0
3 profiles	11046.17	11120.78	.85	.06	.00	0
**4 profiles**	**10811.86**	**10907.79**	**.82**	**.00**	**.00**	**0**
5 profiles	10746.92	10864.17	.84	.24	.00	1
6 profiles	10673.78	10812.36	.83	.16	.00	2

*Note*: Values in bold indicate best-fitting model. AIC = Akaike Information Criteria; BIC = Bayesian Information Criteria; LRT = Vuong-Lo-Mendell-Rubin Likelihood-Ratio Test; BLRT = Bootstrap Likelihood Ratio Test.

**Fig 1:**
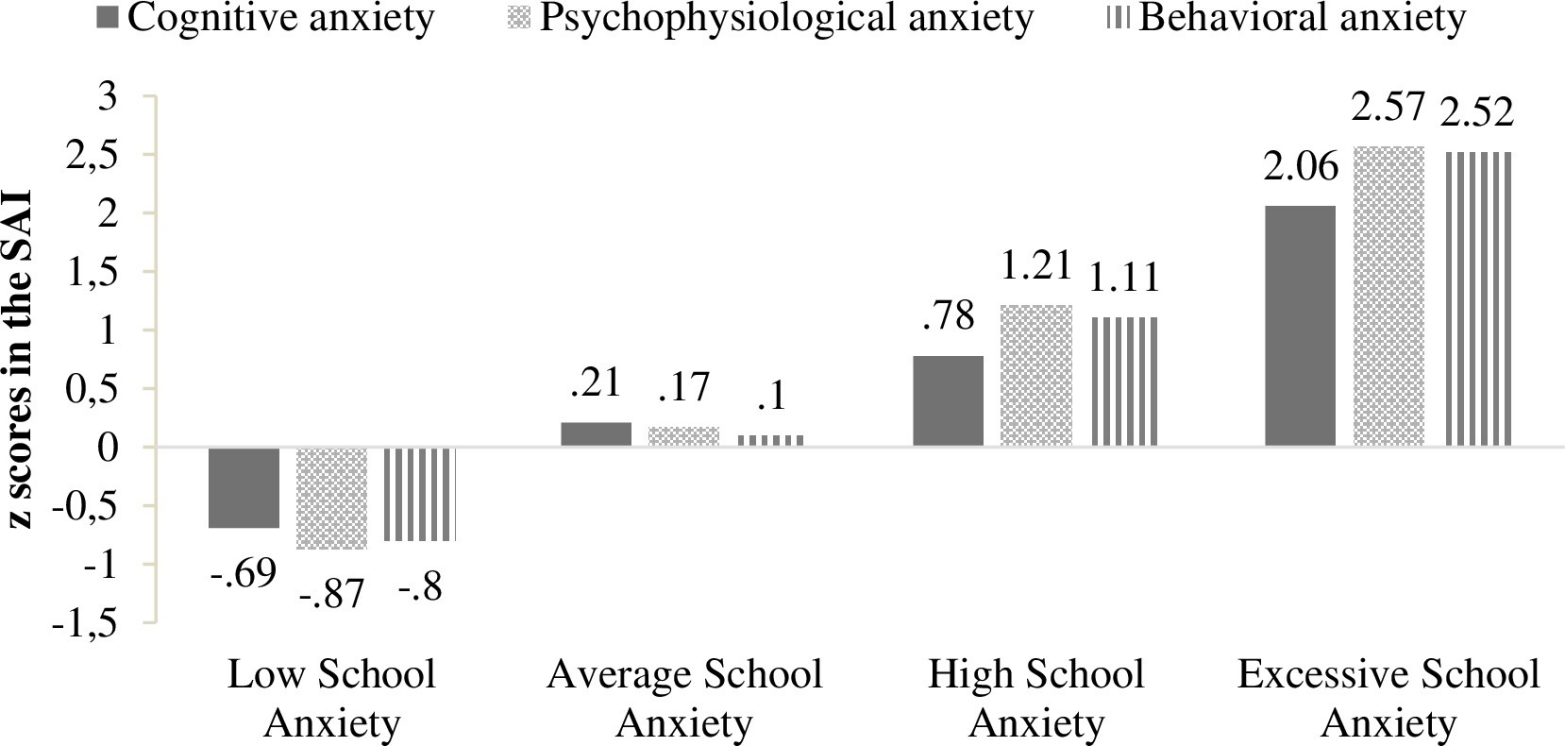
Graphic representation of standardized mean scores for the four-profile model.

In terms of interpretability and psychological significance, the selected solution distinguished four school anxiety profiles. Profile 1 classified 652 students (42.8%) with low scores in the three response systems of anxiety, thus, it was labeled as Low School Anxiety. Profile 2 consisted of 523 students (34.3%) characterized by moderate scores in cognitive anxiety, psychophysiological anxiety, and behavioral anxiety. Therefore, it was named Average School Anxiety. Profile 3 was labeled as High School Anxiety, because its 312 classified participants (20.5%) reported high scores in the three response systems of anxiety. Finally, Profile 4 included 38 individuals (2.5%) who showed very high scores in all anxiety responses, so it was called Excessive School Anxiety. Fig 1 represents the standardized means of the three factors referred to the response systems of anxiety for each latent profile.

### Differences among the school anxiety profiles in psychopathological symptoms

The results of the MANOVA revealed statistically significant differences among the four school anxiety profiles in the nine variables of psychopathological symptoms (Lambda de Wilks = .82, *F*_(27,1521)_ = 11.87; *p* < .001, η^2^ = .07). The students grouped into Excessive School Anxiety showed the highest mean scores in all scales of the SA-45. In other words, this school anxiety profile had significantly higher levels of the nine psychopathological symptoms (referred to the frequency of manifestation of each symptom, as the authors of the SA-45 indicated [28]) than the rest of profiles (see Table 3). In contrast, the participants grouped into Low School Anxiety reported the lowest means in all the variables examined.

**Table 3:** Mean scores, standard deviations, and post hoc contrasts obtained by the school anxiety profiles in psychopathological symptoms.

SA-45 scales	Low School Anxiety		Average School Anxiety		High School Anxiety		Excessive School Anxiety		Statistical Significance and Effect Sizes		
	*M*	*SD*	*M*	*SD*	*M*	*SD*	*M*	*SD*	*F*_*(3*,*1521)*_	*p*	*η* ^*2*^
Depression	5.15	4.18	6.94	4.22	8.52	4.03	10.36	4.51	59.52	< .001	.11
Hostility	5.30	4.36	6.82	4.48	8.02	4.38	9.26	4.64	34.14	< .001	.06
Interpersonal sensitivity	5.07	3.99	6.96	4.04	8.94	3.99	10.10	4.41	77.50	< .001	.13
Somatization	5.51	4.40	7.14	4.17	8.84	4.04	9.57	4.50	50.44	< .001	.09
Anxiety	4.80	3.98	6.99	3.98	8.50	3.79	9.97	4.53	78.91	< .001	.14
Psychoticism	4.52	3.91	6.01	3.90	7.37	3.72	8.44	4.08	46.81	< .001	.09
Obsessive-compulsive	6.61	4.01	8.13	3.91	9.73	3.88	10.34	4.23	50.73	< .001	.09
Phobic anxiety	4.15	4.15	6.01	4.17	8.28	4.23	10.89	4.82	89.05	< .001	.15
Paranoid ideation	5.61	3.85	7.09	3.74	8.44	3.63	9.92	4.16	50.70	< .001	.09

*Note*: SA-45 = Symptom Assessment-45 Questionnaire.

As shown in Table 4, the post hoc tests showed that Excessive School Anxiety scored significantly higher in all scales of the SA-45 in comparison with Average School Anxiety and Low School Anxiety, the effect sizes of these differences being of large and moderate magnitude (*d* = -.54 to -1.25). However, comparisons between Excessive School Anxiety versus High School Anxiety revealed a significant difference only for the phobic anxiety scale and a moderate effect size was found for this difference (*d* = -.61). Moreover, High School Anxiety reported significantly higher scores than Average School Anxiety and Low School Anxiety in the nine psychopathological symptoms. The magnitude of these differences ranged from small to large (*d* = -.27 to -.99). Finally, Average School Anxiety obtained significantly higher scores than Low School Anxiety in the nine variables examined. In this case, small magnitudes were obtained (*d* = -.34 to -.47) except for the anxiety scale, for which the magnitude was moderate (*d* = -.55).

**Table 4:** Cohen’s *d* indices for post hoc contrasts between the mean scores obtained by the school anxiety profiles in psychopathological symptoms.

SA-45 scales	Low School Anxiety vs Average School Anxiety	Low School Anxiety vs High School Anxiety	Low School Anxiety vs Excessive School Anxiety	Average School Anxiety vs High School Anxiety	Average School Anxiety vs Excessive School Anxiety	High School Anxiety vs Excessive School Anxiety
Depression	-.43	-.82	-1.24	-.38	-.81	-
Hostility	-.34	-.62	-.91	-.27	-.54	-
Interpersonal sensitivity	-.47	-.97	-1.25	-.49	-.77	-
Somatization	-.38	-.78	-.92	-.41	-.58	-
Anxiety	-.55	-.94	-1.19	-.39	-.74	-
Psychoticism	-.38	-.74	-1	-.35	-.62	-
Obsessive-compulsive	-.38	-.79	-.93	-.41	-.56	-
Phobic anxiety	-.38	-.99	-1.61	-.54	-1.16	-.61
Paranoid ideation	-.39	-.75	-1.11	-.36	-.75	-

*Note*: SA-45 = Symptom Assessment-45 Questionnaire.

## Discussion

The present study aimed to find out the existence of school anxiety profiles based on Lang’s three-dimensional theory of anxiety and to examine the relationships between these possible groups and psychopathological symptoms (depression, hostility, interpersonal sensitivity, somatization, anxiety, psychoticism, obsessive-compulsive, phobic anxiety, and paranoid ideation) in a sample of Spanish students aged 15 to 18.

The results obtained allow us to confirm the first study hypothesis. Four school anxiety profiles were configured by combining standardized *z* scores in the response system factors. Specifically, students with low, moderate, high, and very high scores in all anxiety responses (i.e., cognitive anxiety, psychophysiological anxiety, and behavioral anxiety) were grouped into the profiles Low School Anxiety, Average School Anxiety, High School Anxiety, and Excessive School Anxiety, respectively. School anxiety profiles based on the three response systems had not been identified before, so this is a contribution to the research of school anxiety on adolescence.

It is important to highlight that the previous study of school anxiety profiles based on anxiety-provoking situations obtained different results [10], even though the participants were from the same country and developmental stage. Spanish adolescents aged 12 to 16 were grouped into three profiles: Low School Anxiety, Average School Anxiety, and High School Anxiety. However, the present work found one more profile: Excessive School Anxiety. This different group may have emerged due to several reasons. In the first place, we used the scores obtained in the three response factors of the SAI, whereas the previous study used the four situational factors of this inventory. In the second place, we used the LPA, whereas the previous work used cluster analysis. LPA is considered a better technique to classify people into homogeneous latent groups than traditional cluster analysis [35], so it can be considered another contribution of the present work. In the third place, there is scientific evidence showing that Spanish adolescents aged 16 to 18 tend to manifest significantly higher levels of cognitive anxiety, psychophysiological anxiety, and behavioral anxiety than their peers aged 14 to 15 [4]. Therefore, the Excessive School Anxiety profile characterized by higher levels of anxiety than the High School Anxiety profile, could have emerged because we recruited older students (15 to 18 years old) than those used in the previous work (12 to 16). Considering these facts, more research on school anxiety profiles throughout adolescence and using LPA is needed to corroborate this four-profile model.

Regarding the differences among the school anxiety profiles in psychopathological symptoms, the Excessive School Anxiety group obtained significantly higher scores in depression, hostility, interpersonal sensitivity, somatization, anxiety, psychoticism, obsessive-compulsive, phobic anxiety, and paranoid ideation (with moderate and large effect sizes) than the profiles Average School Anxiety and Low School Anxiety. These results support the second study hypothesis since the group with the highest scores in the three response systems of school anxiety showed significantly higher levels of all the psychopathological problems examined.

In line with the previous study that used the Spanish version of the SA-45 for examining the relationships of psychopathology with school refusal behavior [22], we have found that adolescents characterized by the highest school anxiety levels showed the worst psychopathological adjustment, which consists of “more somatic complaints (headaches, gastrointestinal issues, etc.), thoughts, feelings, and actions of an obsessive nature, and have a higher risk of negative affect, feelings of inferiority and personal inadequacy, depressive symptoms, anxiety, cognitive distortions and feelings of loneliness” [22] (p. 8). This broadened maladaptive pattern in relation to school anxiety constitutes another contribution of the present work, because so far anxious adolescents had been found to be affected by some common psychiatric problems (i.e., depressive and obsessive-compulsive symptoms, and state and trait anxiety) [16–21]. In this sense, new psychopathological characteristics such as hostility, interpersonal sensitivity, somatization, psychoticism, and paranoid ideation, which have been found in relation to adolescent anxiety in this study, had been identified before in anxious children [36–39] and university students [40–42]. Therefore, these findings have provided important insight into the manifestation of internalizing and externalizing symptoms linked to school anxiety in a less-analyzed educational stage: secondary education.

Regarding the differences between the profiles High School Anxiety and Excessive School Anxiety, interesting results have been found. These groups only differed significantly in phobic anxiety. Specifically, adolescents with Excessive School Anxiety showed significantly higher levels of phobic anxiety with a moderate effect size. The phobic anxiety scale of the SA-45 refers to persistent and irrational anxiety responses to specific situations. Therefore, it makes sense that the response pattern characterized by very high cognitive anxiety, psychophysiological anxiety, and behavioral anxiety belongs particularly to those adolescents with phobic anxiety. In this regard, this finding supports the assumption that the levels of the three response systems of anxiety are higher when people manifest specific phobia [43, 44].

This study has a series of limitations that should be considered in future research. First, these findings have added on to scientific literature the existence of school anxiety profiles based on Lang’s three-dimensional theory among secondary education students. However, the possible school anxiety profiles manifested by primary education or university students remain unknown. Thus, in addition to the replication of the four-profile model in other adolescent samples, LPA should be performed to examine the existence of school anxiety profiles at the other educational stages. In this way, the identification of these profiles would allow the design of prevention programs. Second, the internalizing and externalizing symptoms experienced by the school anxiety groups have been found, but methodology of structural equations or longitudinal data should be used to establish possible causal inferences between school anxiety responses and psychopathological symptoms. Third, it would be useful to look at other variables such as dysfunctional attitudes and beliefs [45], which could explain the development of anxiety responses in the school setting. Finally, future studies should analyze the relationships between psychopathological characteristics and other constructs that may have a negative impact on students’ mental health, as for instance self-concept, since the existing literature on adolescence is scarce [46].

Despite the above-mentioned limitations, relevant practical implications are drawn from our findings for professionals of Health, Psychology, and Education. On the one hand, 20.5% of the Spanish adolescents who participated in the research presented high reactivity in the three response systems of anxiety. Several studies suggest that, in these cases, each anxiety response should be preferably treated with a technique such as self-instructional training for cognitive anxiety, relaxation training for psychophysiological anxiety, and social skills training for behavioral anxiety (see [26] for a review). Moreover, these adolescents grouped into the High School Anxiety profile need specific training to reduce psychopathology and foster their mental health and well-being. In these sense, previous works found that self-compassion training and positive psychology school-based interventions significantly reduce psychopathological symptoms such as anxiety, depression, distress, or interpersonal sensitivity, whereas enhance positive mental states such as self-esteem or optimism [47, 48]. In addition, all members of the school community should fully participate in the implementation of these programs to guide adolescents in their progress and to promote a positive school climate [49, 50].

On the other hand, special attention must be paid to adolescents with Excessive School Anxiety and to the high severity of psychopathology that they manifest. Thus, 2.5% of the sample showed very high levels of cognitive, psychophysiological, and behavioral anxiety. It is important to highlight that the psychophysiological and behavioral components predominated in this group of students, with standardized means over 2.5 for both response systems. In this regard, adolescents grouped into Excessive School Anxiety should receive techniques of treatment specially focused on reducing psychophysiological responses such as muscle tension or respiratory rate, and behavioral responses like avoidance or motor restlessness [51, 52]. Furthermore, these students, in comparison with those grouped into High School Anxiety, have the same severity of eight in nine psychopathological symptoms (i.e., depression, hostility, interpersonal sensitivity, somatization, anxiety, psychoticism, obsessive-compulsive, and paranoid ideation) and are characterized by higher phobic anxiety. This last aspect implies that adolescents with Excessive School Anxiety tend to consider future specific situations as dangerous (e.g., feeling afraid to go out of their house alone), these predictions being profuse and unrealistic.

Due to all the above factors, applying mindfulness meditation could benefit students who show the Excessive School Anxiety profile. It has proved to train adolescents to be aware of present-moment experience and predictive thoughts, and to develop acceptance and emotion regulation instead of judging their thoughts and actions [53]. Thus, this risk profile could learn to relax the nervous system, to control their impulsive responses of avoidance and to have a realistic perception of those specific situations that provoke anxiety. Finally, mindfulness meditation should integrate positive psychology to foster optimistic thoughts, which could reduce depression, hostility, and the rest of psychopathological symptoms experienced [54].

In conclusion, this study adds on the scientific literature the existence of four school anxiety profiles in the Spanish adolescent population, which are based on Lang’s model of the triple response system. Students who showed high and very high reactivity in the three response systems (i.e., cognitive, psychophysiological, and behavioral anxiety) were characterized by experiencing high severity of psychopathological symptoms. Adolescents with a very high reactivity in the three anxiety response systems were particularly vulnerable to phobic anxiety. In this sense, it is necessary to extend the identification of school anxiety profiles to other educational stages and to find the possible causal relationship between school anxiety and psychopathology for a better comprehension of students’ needs.

## Supporting information

S1 File Data set of school anxiety and psychopathological symptoms. (SAV)
